# New hope for eradication of HIV from the body: the role of polymeric nanomedicines in HIV/AIDS pharmacotherapy

**DOI:** 10.1186/1477-3155-12-9

**Published:** 2014-03-22

**Authors:** Jimma Likisa Lenjisa, Minyahil Alebachew Woldu, Gizaw Dabessa Satessa

**Affiliations:** 1 Department of Clinical Pharmacy, Ambo University, College of Medicine and Health Sciences, P.O. Box 19, Ambo, Ethiopia; 2 Department of Pharmacology, Ambo University, College of Medicine and Health Sciences, Ambo, Ethiopia

**Keywords:** HIV/AIDS, Antiretrovirals, Nano-polymers, Nanomedicines

## Abstract

Human immunodeficiency virus continued to be the greatest challenge and killer disease of the 21st century despite the advent of potent highly active antiretroviral therapy which are limited by their severe adverse effects, significant drug interactions, frequent dosing, limited bioavailability, and less access to viral reservoir sites like macrophages. Nano-medicines are becoming new hopes in avoiding these shortcomings of conventional antiretroviral drugs. The emphasis of this review is mainly the application of polymers based nanomedicines in pharmacotherapy of HIV/AIDS. Most of the studies to date on this area are *in vitro* and human clinical trials are totally missed. However, many interesting points are uncovered through this review like the possibility of achieving high intracellular concentration of drugs, very good antiretroviral activity, improved bioavailability, reduced toxicity and release of the drugs from nanocarriers for long time reducing the need for frequent dosing. Indeed, a lot of assignments left behind for researchers to overcome the challenges hindering the wider application of nanomedicines in treatment of HIV/AIDS.

## Introduction

Nano-medicine is a term implying the application of nanotechnology (the technology that uses nanosized particles) for therapy and diagnosis of diseases. Nanoparticles have improved pharmacokinetics and tissue distribution of therapeutic agents there by diminishing toxicity by their preferential accumulation at the target site. In addition, they improve therapeutic potential of drugs by facilitating intracellular delivery and prolonging their retention time either inside the cell or in blood circulation [1,2].

Nanoparticles can be categorized into three groups: Inorganic nanoparticles, solid lipid nanoparticles and polymer nanoparticles. Inorganic nanoparticles are the generic term for several nanoparticles such as gold and magnetic nanoparticles. Solid lipid nanoparticles combine the advantages but avoiding the disadvantages of other colloidal carriers such as emulsions, liposomes and polymeric microparticles and nanoparticles. Polymer nanoparticles involve various natural or biocompatible synthetic polymers [1,3,4].

Polymer therapeutics is a family of compounds and drug delivery technologies that uses water-soluble polymers as a common core component. One of the biggest advantages of using these polymers in drug delivery is the capability to manipulate their properties such as molecular weight, linkers and many more to adapt to the drug delivery requirements. They can be subdivided into several groups: polymer drugs, polymeric –protein conjugates, polymer drug conjugates, multicomponent polyp lexes being developed as non-viral vectors, polymer micelles and dendrimers [2,3].

Polymer-protein conjugates using polyethylene glycol (PEG) called PEGylation as polymer component are currently the most advanced class of polymer therapeutics. They are developed to avoid some of the drawbacks of peptide, protein and antibody-based drugs like a short half-life, poor stability and solubility, increased clearance by the reticuloendothelial system (RES) and immunogenicity. PEGylation has been employed in treatment of viral hepatitis as well as different types of malignancies [4,5].

Polymer-drug conjugates are becoming a fast growing field, where nearly a dozen of polymeric conjugates advancing to the clinical trial stage. Several advantages of these technologies are being reported over the corresponding parent drugs by different clinical trials including fewer side effects, enhanced therapeutic efficacy, ease of drug administration, and improved patient compliance. The therapeutic efficacy of these agents is increased primarily through an enhanced permeability and retention (EPR) effect of long-circulating polymers [5].

Dendrimers are nanostructures obtained from macromolecules such as polyamidoamine (PAMAM), and polypropyleneimin; and are highly branched with an inner core. They are unique in that they have series of branches, multivalences, well defined molecular weight and globular structure with controlled surface functionality. The presence of multivalency nature enables the attachment of several drug molecules, targeting groups and solublising groups onto their surfaces in a well defined manner. Beside their use in drug delivery, dendrimers are also pharmaceutically active compounds against some diseases [4,6].

Micelles are formed from amphiphilic surfactants which spontaneously associate in aqueous medium to form core (hydrophobic)-shell (hydrophilic) structures or vesicles. Polymeric micelles are able to reach parts of the body that are poorly accessible. However, their targeting ability is limited by the low drug loading and low drug incorporation stability leading to the loaded drug to be released before getting to the site of action [7,8].

In general, nanomedicines particularly polymer based drug delivery systems are highly fascinating and hence attracting the attention of scientists from different corners of the world. This is especially true for the diagnosis, prevention and treatment of intracellular infections like hepatitis, tuberculosis and HIV/AIDS [9] which can be considered as a triple plague in developing countries like Ethiopia. The underlined fact for this is that we can easily manipulate their properties like molecular weight to adapt to the drug delivery requirements. This review, however, focuses on the role of polymer based nanomedicines in the prevention and treatment of HIV/AIDS with the intention of finding out drugs with better efficacy and toxicity profile which can also completely eradicate the virus from the body.

### The complexity of HIV/AIDS as a disease: challenges to complete eradication of the virus from the body

If left untreated, HIV infection is associated with very high viral load in the body leading to progressive fall in immune cells particularly CD4+ T cells. This can be interrupted by treatment with highly active antiretroviral therapy (HAART), which should contain at least three drugs regimens made up of at least two classes of antiretroviral agents. However, the great challenge is that immediately after initial infection, this virus is able to establish reservoirs where it escapes from the effect of drugs and keeps releasing the viral progeny to the blood as long as the patient lives. This makes it one of the chronic and lifelong diseases especially with the introduction of HAART. There are two types of viral reservoirs within tissues that serve this role. The anatomical reservoirs; these are tissues inaccessible to optimal levels of antiviral drugs due to due to the presence of barriers, such as the blood–brain barrier (BBB), blood-cerebrospinal fluid barrier, and blood-testes barrier. and the others are cellular reservoirs; cells in which this virus remains latent and hence escaping the action of antivirals due to the presence of efflux proteins such as P-glycoprotein and multidrug resistance protein on the cell surface preventing the drugs from attaining therapeutic intracellular concentrations [10,11].

Dendritic cells within lymphoid tissue trap a large number of extracellular virions on their surface to protect virus from antiretroviral drugs. On top of this, latently infected CD4+ T cells help the HIV to persist despite the presence of effective antiretroviral therapy as it is not replicating at this stage. Last but not least, monocytes/macrophages that are specifically found in brain, pulmonary alveoli, spleen and lymph nodes are relatively long-lived cells since HIV has very low cytopathic effects on them making them a persistent reservoir of HIV regardless of the presence of highly active antiretroviral therapy [11-13]. Therefore, it is the existence of these persistent and stable reservoirs for the virus that makes it difficult to efficiently eradicate HIV from the body even with the advent of HAART. This is due to the fact that these drugs in free form have poor local bioavailability and low residence time in these reservoirs when administered systemically [14] highlighting the need for new drug or delivery system with the potential of averting these problems so as to achieve a cure from HIV/AIDS.

### The need to advance HIV/AIDS clinical therapies

The HIV/AIDS epidemic is one of the major public health threats especially in sub-Sahara countries. Globally, about 35.3 million people were living with HIV in 2012 which is an increase from previous years as more people are receiving the life-saving antiretroviral therapy. The prevalence of HIV/AIDS continues to increase and it is expected that over 90 million people will ultimately be infected in Africa alone. On the other hand, there were 2.3 million new HIV infections and 1.6 million AIDS deaths in 2012 globally. In 2012, 9.7 million people in low- and middle-income countries received antiretroviral therapy, representing 61% of all who were eligible under the 2010 World Health Organization (WHO) HIV treatment guidelines. However, under the 2013 WHO guidelines, the HIV treatment coverage in low- and middle-income countries represented only 34% of the 28.3 million people eligible in 2013. Antiretroviral therapy not only prevents AIDS-related illness and death: it also has the potential to significantly reduce the risk of HIV transmission and the spread of tuberculosis. From 1996 to 2012, antiretroviral therapy averted 6.3 million AIDS-related deaths worldwide, including 5.2 million deaths in low- and middle-income countries [15].

However, despite the clear advantages of HAART in management and prevent of HIV, there are several significant shortcomings. Significant drug interactions, additional and or synergistic toxicity as a result of combination, low adherence rate secondary to pill burden and frequent dosing, and potential for development of drug resistant virus which is even more difficult to treat. An additional important issue is that upon discontinuation of treatment or when resistance develops, even with HAART, the viral load rebounds in the blood [16]. Lastly, systemically available drug needs to cross biological barriers for delivery to cellular and anatomical sites which most currently available antiretroviral formulations couldn’t. CNS availability of most anti-retroviral agent is very low due to poor permeability across the blood–brain barrier. The consequence of these interdependent processes is insufficient concentrations and very short residence time of the anti-retroviral agents at the cellular and anatomical sites [16,17].

### Nanotechnology approaches for delivery of antiretroviral drugs in HIV/AIDS management

Targeted delivery to HIV reservoir sites would be of significant benefit because many antiretroviral drugs do not penetrate these sites optimally which contribute not only to viral persistence, but also to the development of drug resistance [18,19].

Li Wan and colleagues had investigated the peritoneal macrophage up take, pharmacokinetics and biodistribution of Macrophage-Targeted PEG-fMLF (*N*-Formyl- Methionyl-Leucyl-Phenylalanine) nanocarriers for improving HIV drug delivery. They showed that uptake by macrophages increased many folds, and also accumulation of nanocarriers into macrophages of liver, kidneys and spleen increased in similar manner. This implies that by covalent conjugation of macrophage targeting moiety fMLF to PEG, it is possible to achieve a higher accumulation in macrophages residing in tissues [20]. However, the reduction of interaction with cell-surfaces due to PEG limits its use as a biomaterial for cell-surface or intracellular drug delivery and targeting. Therefore, the very property of PEGylation that has made it clinically useful and commercially successful also limits its application for drug/nanocarrier targeting to HIV reservoirs or sanctuary sites such as macrophages, and the central nervous system [21].

Regardless of this limitation, however, PEG based nano carriers had been investigated for delivery of antiretroviral to the brain in US. In this study, they developed and evaluated nanocarriers consisting of PEG- or Pluronic-PEI biodegradable networks, star PEG-PEI, or PAMAM-PEI-PEG dendritic networks, as well as nanogels decorated with multiple ApoE peptide molecules, specifically binding to the apolipoprotein E receptor. It was shown that there is efficient uptake of the drugs by monocyte-derived macrophages and there was minimal cytotoxicity. The presence of core-shell and decoration with proteins like brain ApoE peptide molecules even gives it the highest efficacy against the virus in the CNS macrophages [22].

Another study done on the application of PEG for delivery of antiretroviral drugs also reported the beneficial effects of this nanopolymer. Accordingly, when PEG of various length was conjugated with an antithrombin-binding carrier pentasaccharide (CP) for delivery of enfuvertide, it shows several fold increase in half life enabling weekly dosing of the drugs as well as reducing the adverse effects associated with the drug. This indicates that the designed conjugate is a promising compound with strong potency as a novel long-lasting anti-HIV-1 drug [23].

The recent *in vitro* study of Sumit and co-workers developed, evaluated, and compared colloidal gold-loaded, poly (d,l-lactic-co-glycolic acid)-based nanoparticles containing antiretroviral drug stavudine and uptake of these nanoparticles by macrophages. It is known that Stavudine had been one of the first line antiretroviral drugs until the release of the 2010 ART WHO guideline for adult and adolescents in resource limited settings. However, the drug had been out of use in adult people living with HIV because of serious toxicity associated with it. Now it seems that there is a hope that this drug may back to the market as a result of the advent of novel drug delivery technologies like nanomedicines. In this regard, study in India found that stavudine was released from polymeric nanoparticles for a prolonged period (over 63 days) and up taken by macrophages and hence the promising opportunity for this drug to enjoy the market again. In addition to this, even if the finding must be confirmed by *in vivo* studies, the fact that this drug could be administered in low dose as well as targeted to specific cell in this study may minimize the systemic toxicity of the drug several folds [24].

Other potential and fascinating nano polymers for delivery of antiretroviral drugs are dendrimeres. They are polymers with large amounts of peripheral groups and interior cavities making them potential vectors for chemical drugs, peptides and genes for HIV inhibition. These materials are capable to either interacting with the peripheral groups or be encapsulated into the cavities of dendrimers. More surprisingly, many recent studies showed that these polymers have very good antiviral activity themselves against HIV in different animal model beside their role as a nanocarrier for delivery of antiretroviral drugs [25-28].

The team of Avrim investigated the role of nano polymers for transdermal delivery of zidovudine which is the oldest and first line back bone drug in resource limited settings. They showed that Eudragit RL100 based delivery of zidovudine achieved the highest in vitro cumulative release profile giving the closest results with the free drug. This could be attributed to the hydrophilic nature and swelling of ERL polymer. In contrast, drug release from hydrophobic Ethyl Cellulose film was slow, as the polymer was not swellable [29].

In other studies, efavirenz-loaded micelles using surface aptamers to target CD4 cells have been produced in an oral solution for pediatrics [30,31]. In rats model, the efavirenz-loaded micellar formulation was compared to that of a suspension prepared with the content of efavirenz capsules in 1.5% carboxymethylcellulose solution and an EFV solution in a medium-chain triglyceride. This formulation showed that the encapsulation of efavirenz into polymeric micelles of different poly(ethylene oxide)–poly(propylene oxide) block copolymers significantly improves oral bioavailability and reduces the interindividual variability [32].

Monotherapy in HIV/AIDS management is ineffective and also associated with highest degree of antiretroviral drug resistance which if develops is difficult to treat. For this reason treatment of HIV/AIDS today relies on the combination of at least three drugs from at least two pharmacologic classes of drugs. There are many studies done on delivery of single antiretroviral drug using nano polymers targeting less accessible tissues to free drugs whereas studies on delivery of several antiretroviral drugs in a single nano polymer is rare.

The first study which demonstrated the possibility of nanoparticles [Poly (DL-lactide-co-glycolide) nanoparticles (PLGA-NPs)] delivery with a combination of three antiretroviral drugs in single nanoparticle was that of Destache and co-workers. It was showed that nanoparticles based delivery of single dose of lopinavir/ritonavir and efavirenz resulted in better and sustained suppression of the HIV-1 from both serum and tissues (viral reservoirs and brain) for at least a month compared to free drugs administration in mice model. This seems permanent solution for the high non-adherence rate which in turn lead to resistant but more difficult to treat virus; hence treatment failure. The same study showed parenteral delivery of antiretroviral drugs is possible and hence overcome the issue of absorption and metabolism with the resultant improvement in bioavailability these drugs.

This finding created very good opportunity for future researchers to design delivery of highly active antiretroviral therapy using nano-polymers technology which is very significant breakthrough in HIV/AIDS therapy [33].

In another study, nanoparticles for delivery of zidovudine-lamivudine combination was formulated and characterized by Sankara V and co-workers. Nanoparticles of AZT-3TC were prepared through emulsion polymerization in a continuous aqueous phase of different polymers such as poly (lactic-co-glycolic acid) PLGA(50:50), poly(lactic acid), and poly(methyl methacrylate) PMMA, methyl methacrylate- sulfopropylmethacrylate (MMA-SPM). It was observed that *in vitro* drug release was higher from PLGA compared to the other nanoparticles for both drugs. For both drugs, release from PLGA nanoparticles was greater than 95% within 10 hours and acute toxicity to animal cells was not detected [34].

Very latest study of Annemarie and co-workers investigated delivery of three antiretrovirals combination in single nano-polymers similar to the work of Destache and co-workers. In this work, they developed nanoparticles of biodegradable polymer, poly-(dl-lactide-coglycolic acid; PLGA) containing efavirenz (EFV) and boosted lopinavir (lopinavir/ritonavir; LPV/r) by a highpressure homogenization method. The investigators of this study demonstrated the significant uptake of the combination of antiretroviral drugs particles compared to the soluble free antiretroviral drugs, the subsequent release of levels of antiretroviral drugs in the nuclear, cytoskeleton, and membrane fractions of cells with no toxicity for 28 days [35].

### Challenges to widely apply nanomedicine for HIV/AIDS therapy

Even though, nanomedicines are the promising future of HIV/AIDS prevention and treatment, several hurdles remain unresolved, including but not limited to toxicity, unwanted biological interactions and the difficulty and cost of large-scale synthesis of nanopharmaceuticals [19]. Another challenge is the fact that targeted delivery of antiretroviral drugs using nano polymers to viral reservoir sites may lead to HIV drug resistance. This could be because of two reasons. Firstly, the targeted delivery of an antiretroviral drugs which if not accompanied by systemic HAART administration will lead to suboptimal doses of the drug in non-targeted tissues, with the potential to select out drug resistant mutations there.

Furthermore, most studies involving nanocarriers use a single antiretroviral drug, which would effectively select out resistant virus in targeted tissues [29,36]. highlighting the need to combine at least three drugs for use with nanocarriers as in conventional HAART in future researches.

## Conclusions

In this review, we have gone through many works done on the application of nano-polymers for the treatment and prevention of HIV infections. This paper showed that nanopolymers of different quality are designed and evaluated for delivery of ARVs. Of all, surprising thing is the fact that some polymers like dedrimers have an inherent antiviral activity beside their carrier roles which further makes them more appropriate to use them for delivery of ARVs in medicine.

In this paper, we have also discovered very promising features for HIV therapy such as improving residence time of the drugs at the targeted sites, several fold increase in the uptake of the drugs to those previously less accessible viral reservoir tissues, improved antiviral efficacy and significantly reduced toxicity of antiretroviral drugs. Moreover, we have observed that, through application of nanomedicines, there will be the possibility of dosing ARVs every month or more intervals tackling the current problems of non-adherence related to frequent and larger dose administration of conventional formulations. Not only these but also there will be possibility of designing sustained release and controlled release ARV regimens to be delivered through transdermal, subcutaneous and intramuscular routes enabling the drugs to bypass first pass effect. These findings clearly suggest the hope that HIV can be completely eradicated from the body through the application of nano-polymers for delivery of ARVs in the near future.

However, a lot of assignments left behind for researchers to overcome the challenges hindering the wider application of nanomedicines in treatment of HIV/AIDS. Beside this, clinical trials involving the use of nano-polymers to deliver HAART regimens should be designed to investigate both the beneficial and drawbacks of this technology.

## Competing interests

The authors declare that they have no competing interests.

## Authors’ contributions

JLL has searched the important literatures, and drafted the manuscript. MAW has also searched the important literatures, and drafted the manuscript. Likewise GDS has searched the important literatures, and drafted the manuscript. Finally, all authors read and approved the final manuscript.
